# Does circulating progesterone mediate the associations of single nucleotide polymorphisms in progesterone receptor (PGR)-related genes with mammographic breast density in premenopausal women?

**DOI:** 10.1007/s12672-021-00438-1

**Published:** 2021-11-03

**Authors:** Favour A. Akinjiyan, Yunan Han, Jingqin Luo, Adetunji T. Toriola

**Affiliations:** 1 Medical Scientist Training Program, Washington University School of Medicine, St. Louis, MO 63110 USA; 2 Division of Public Health Sciences, Department of Surgery, Washington University School of Medicine, Campus Box 8100, 660 South Euclid Ave, St. Louis, MO 63110 USA; 3 Department of Breast Surgery, First Hospital of China Medical University, Shenyang, 110001 Liaoning Province China; 4 Alvin J. Siteman Cancer Center at Barnes-Jewish Hospital and Washington University School of Medicine, St. Louis, MO 63110 USA

**Keywords:** Progesterone, Genetics, Mammographic breast density, SNPs, Breast, Premenopausal

## Abstract

Progesterone is a proliferative hormone in the breast but the associations of genetic variations in progesterone-regulated pathways with mammographic breast density (MD) in premenopausal women and whether these associations are mediated through circulating progesterone are not clearly defined. We, therefore, investigated these associations in 364 premenopausal women with a median age of 44 years. We sequenced 179 progesterone receptor (PGR)-related single nucleotide polymorphisms (SNPs). We measured volumetric percent density (VPD) and non-dense volume (NDV) using Volpara. Linear regression models were fit on circulating progesterone or VPD/NDV separately. We performed mediation analysis to evaluate whether the effect of a SNP on VPD/NDV is mediated through circulating progesterone. All analyses were adjusted for confounders, phase of menstrual cycle and the Benjamini–Hochberg false discovery (FDR) adjusted p-value was applied to correct for multiple testing. In multivariable analyses, only PGR rs657516 had a direct effect on VPD (averaged direct effect estimate = − 0.20, 95%CI = − 0.38 ~ − 0.04, p-value = 0.02) but this was not statistically significant after FDR correction and the effect was not mediated by circulating progesterone (mediation effect averaged across the two genotypes = 0.01, 95%CI = − 0.02 ~ 0.03, p-value = 0.70). Five SNPs (PGR rs11571241, rs11571239, rs1824128, rs11571150, PGRMC1 rs41294894) were associated with circulating progesterone but these were not statistically significant after FDR correction. SNPs in PGR-related genes were not associated with VPD, NDV and circulating progesterone did not mediate the associations, suggesting that the effects, if any, of these SNPs on MD are independent of circulating progesterone.

**Supplementary Information:**

The online version contains supplementary material available at 10.1007/s12672-021-00438-1.

## Introduction

Mammographic breast density (MD), which refers to the amount of epithelial and stromal tissues in relation to adipose tissue in the breast [1] is a strong risk factor for breast cancer with odds ratio as high as 4–6 for women with > 75% dense tissue in their breasts compared to those with < 5% dense tissue [2, 3]. Progesterone regulates breast development [4, 5] and can cause proliferation within the breasts [6]. In preclinical models, estrogen/progesterone stimulates expansion of the mammary stem and progenitor cells, generating denser mammary morphology [7]. Studies have demonstrated that progesterone is associated with MD and breast cancer risk [8–13]. In the Women’s Health Initiative trial, 12 months of estrogen/progestin use was associated with a 5% increase in MD [14], and subsequent follow-up indicated higher breast cancer incidence in women on estrogen/progestin [15].

Progesterone exerts its functions by binding to the progesterone receptor (PGR), a nuclear receptor which, when activated, results in changes in gene expression of various proliferative genes [16, 17]. Single nucleotide polymorphisms (SNPs) in the PGR gene modulate changes in MD in response to menopausal hormone therapy. However, there are conflicting results on the associations of genetic variations in progesterone-related pathways with MD [18–20]. In addition, there are limited data in premenopausal women [19], where hormonal variation during menstrual cycle makes it challenging to evaluate these associations. Nonetheless, it is important to investigate the associations of SNPs in the PGR gene with MD in premenopausal women as this could identify biological pathways that can be targeted in breast cancer prevention in younger women. Further, although the associations of SNPs in the PGR gene with MD could be primary i.e. directly through modulation of gene expression, or indirect, mediated by circulating progesterone levels, to the best of our knowledge, no studies have investigated these potential mediating effects. Finally, there are limited studies on how determinants of MD influence PGR SNPs. Understanding these could provide additional insight into how PGR SNPs are associated with MD. Our objectives in this study are threefold: (i) to investigate the associations of genetic variations in PGR and PGR-related SNPs (PGR Membrane Component 1 (PGRMC1) with MD in premenopausal women; (ii) determine whether these associations are mediated by circulating progesterone levels; (iii) evaluate the associations of PGR and PGR-related SNPs with known determinants of MD (such as body mass index).

## Materials and methods

### Study population

**Fig. 1:**
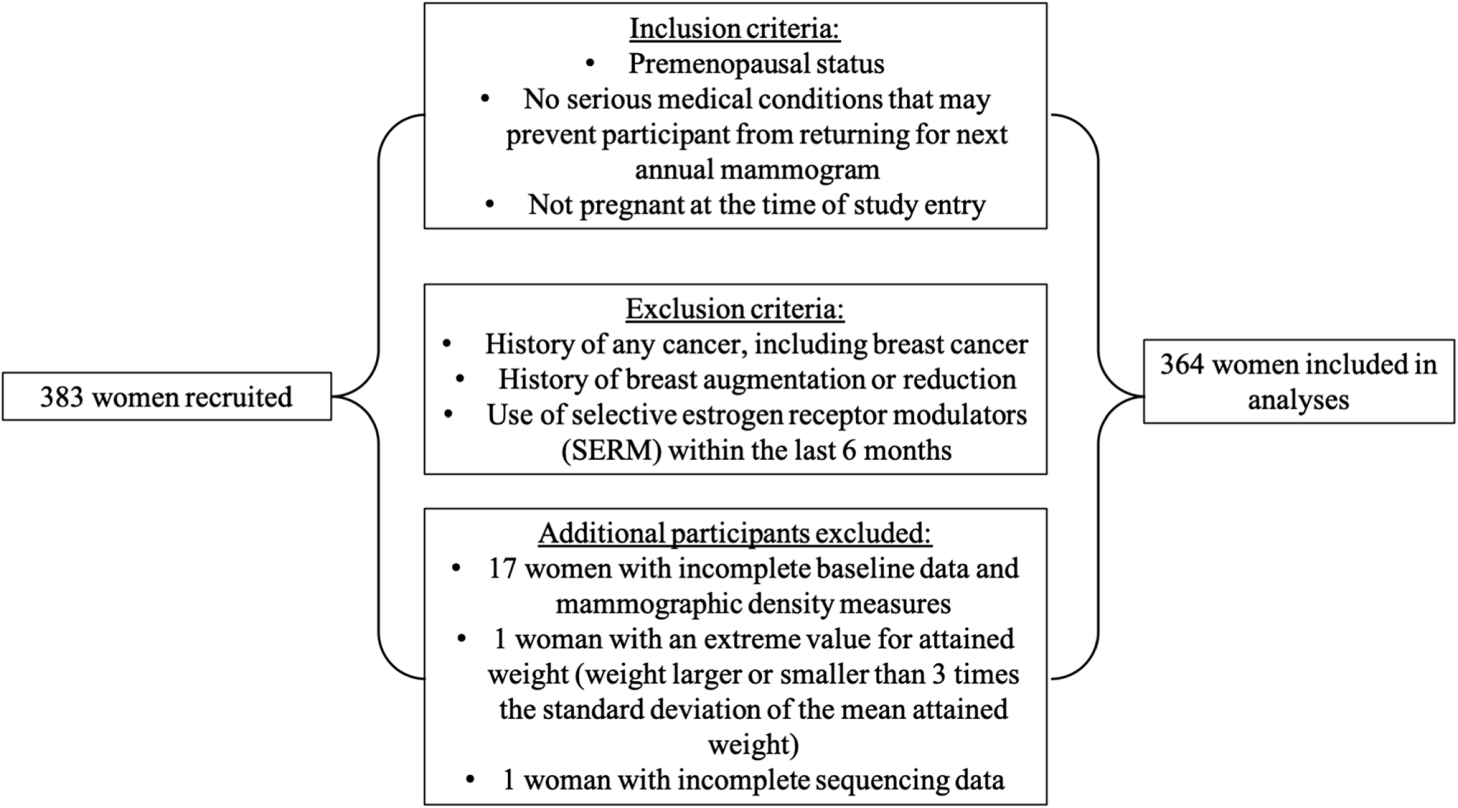
Schematic depicting inclusion and exclusion criteria for study participants

We recruited 383 healthy premenopausal women who were scheduled for an annual screening mammogram at the Joanne Knight Breast Health Center at Siteman Cancer Center at Washington University School of Medicine, (St. Louis, MO) in 2016. Our previous work provides a detailed description of the study population [21]. In brief, premenopausal women who were scheduled for their annual screening mammography at the BHC were mailed study flyers by research coordinators two weeks to one month in advance. Follow-up calls were then within 7 days of the scheduled appointments to screen interested individuals, provide further details and answer questions on the study [21]. Inclusion criteria include (i) premenopausal status. We identified women as premenopausal if they had a regular menstrual period within the preceding 12 months, no prior history of bilateral oophorectomy, and not used menopausal hormone therapy, (ii) No serious medical conditions that could prevent participant from returning for annual mammogram in 12 months, and (iii) not pregnant at the time of study entry. Women with previous cancer history, previous breast surgery and use of selective estrogen receptor modulators (SERM) within the last 6 months were excluded (Fig. 1).

Study participants were asked to fast on the day of their annual screening mammogram. On the day of the mammogram, blood samples were collected from each participant within a few hours of their mammogram. Height and weight measurements were collected after mammogram using a stadiometer and OMRON Full Body Sensor Body Composition Monitor and Scale model HBF-514C, respectively. Blood samples were stored at -80℃ within 30 min of collection at the Tissue Procurement Core at the Siteman Cancer Center. Participants also completed a questionnaire on determinants of MD and risk factors for breast cancer. All participants provided informed consent. The study was approved by the Institutional Review Board of the Washington University School of Medicine.

### Single nucleotide polymorphisms in progesterone receptor (PGR)-related genes

Sequencing was performed at the McDonald Genome Institute, Washington University. Automated dual indexed libraries were constructed with 100-250 ng of genomic DNA utilizing the KAPA HTP Library Kit (KAPA Biosystems) on the SciClone NGS instrument (Perkin Elmer) targeting 250 bp inserts. 365 women had genomic DNA yield of at least 100, from which libraries were pooled at an equimolar ratio yielding ~ 5 µg per library pool prior to the hybrid capture. Library pools were hybridized with an IDT xGen Predesigned Gene Capture Pool (Integrated DNA Technologies) covering 32 genes of the human genome. The concentration of each captured library pool was accurately determined through qPCR (Kapa Biosystems) to produce cluster counts appropriate for the HiSeq4000 platform (Illumina). 2 × 151 bp paired end sequence data was produced according to Illumina manufacturer specification to generate approximately 1–2 Gb per IDT Targeted Capture sample, ensuring > 95% of the targets covered at a minimum of 500 × depth of coverage. Variants were called using GATK, annotated by ENSEMBLE VEF and stored in variant call format. snpEff (version 4.0, build 2014–09-13) was used to further annotate the variants using human genome build 37 [22]. The R package “vcfR” was utilized to read in data to extract genotype and relevant quality measures [23]. Complete sequencing data was available for 364 women, who were included in the current analyses.

### Circulating progesterone levels

Circulating progesterone levels were assayed at the Department of Laboratory Medicine, Boston Children’s Hospital. Progesterone levels were assayed using a competitive electrochemiluminescence immunoassay on the Roche E Modular system (Roche Diagnostics, Indianapolis, IN). The assay is FDA-approved for clinical use and has been used in previous studies [24, 25]. The intra-assay coefficients of variation were 2.9% at 0.73 ng/mL and 0.9% at 32.4 ng/mL based on blind replicates in our samples. The inter-assay coefficients of variation were 4.8% at 0.73 ng/mL and 2.0% at 35.3 ng/mL. Sensitivity was 0.15 ng/mL and specificity was 0.81 for the immunoassay.

### Mammographic breast density assessment

We used Volpara version 1.5 (Matakina Technology Ltd) to determine volumetric measures of MD, and we focused on volumetric percent density (VPD) and non-dense volume (NDV) in this study. VPD is the ratio of the volume of fibroglandular tissue (i.e., dense volume) to the total breast volume, expressed as a percentage. Volpara uses a computerized algorithm that calculates the X-ray attenuation at each pixel and converts the attenuation to an estimate of the tissue composition to create a density map [21, 26, 27] and averages the cranial–caudal and mediolateral oblique views of the left and right breasts [28, 29]. Corresponding to the four categories [(a)–(d)] of the Breast Imaging Reporting and data System (BI-RADS; 5th edition) [30, 31], Volpara VPD measures translate to: < 3.5% (a, almost entirely fatty breasts); 3.5–7.5% (b, scattered areas of fibroglandular density); 7.5–15.5% (c, heterogeneously dense breasts); 15.5–34.5% (d, extremely dense breasts) [21, 32].

### Statistical analyses

We sequenced 179 SNPs in PGR-related pathways. The SNPs were subject to quality control, removal of synonymous variants, filtering to keep SNPs with 10 or more samples each having at least 5 reads. Each SNP was coded using the dominant mode of inheritance into genotype = 1 in the presence of at least one minor allele and genotype = 0 in the presence of two of the major alleles. Genotype counts and percentages were summarized for each SNP. The associations between SNPs and between a SNP and categorical variables were investigated using Fisher’s exact test. Circulating progesterone, VPD and NDV were analyzed in logarithm scale to ensure normality. The variables included in multivariable analyses were: (i) age (continuous), (ii) current BMI (continuous), (iii) family breast cancer history (Yes/No), (iv) body shape at age 10 based on the Stunkard [33] 9-level figure pictogram categorized into 4 groups: body size 1 or 2, body size 3 or 4, body size 5, and body size 6 or higher, (v) composite score of age at 1st birth and parity (age at 1st birth < 25 and 3 or more kids vs. age at 1st birth ≥ 25 and 3 or more kids vs. age at 1st birth ≥ 30 and 1 or 2 kids vs. age at 1st birth in 25 ~ 29 and 1 ~ 2 kids vs. nulliparous), (vi) phase of menstrual cycle (luteal vs. secretory vs. unknown).

For direct associations of SNPs with quantitative circulating progesterone and MD, linear regression models were fit on circulating progesterone or MD separately, with a SNP alone or additionally with incorporation of the six covariates. The linear coefficient estimate associated standard error (SE) and Wald test P value on the SNP were reported from the linear models. Wilcoxon test was also used to test the difference in circulating progesterone and MD by genotype, which yielded similar results to fitting linear regression. For indirect association analysis, the mediation analysis was performed to evaluate the potential mediator role of circulating progesterone, i.e., SNP effecting on MD through circulating progesterone. The mediator model in the mediation analysis fits circulating progesterone on a SNP to estimate the effect of a SNP on circulating progesterone, with inclusion of the six covariates. The outcome model in the mediation analysis, while adjusting for covariates, fits MD on circulating progesterone, a SNP under analysis and the interaction of circulating progesterone and SNP in consideration of potentially differing effect of genotypes to examine homogeneity/heterogeneity between genotypes. We report from the mediation analysis: (i) the averaged direct effect (ADE) which was estimated as the coefficient of a SNP for each genotype in the outcome model, (ii) the averaged causal mediation effect (ACME) which was estimated as the coefficient of mediator circulating progesterone in the outcome model multiplying the coefficient of a SNP in the mediator model, and (iii) the total effect which was essentially the sum of ADE and ACME, all accompanied with 95% confidence interval (CI) and p-value. For single SNP association analyses, we focused on SNPs with a genotype = 1 count ≥ 10 and frequency of 3% or higher.

The SKAT [34] (sequence kernel association test, with default linear weighted kernel and weight function) approach was used to investigate the overall gene set/pathway significance integrating SNPs on the PGR gene and the PGRMC1 gene, alone and collectively representing the PGR pathway, without and with inclusion of the six covariates. SNPs with high missing rate (SKAT default = 15%) were excluded from analyses while other SNPs with lower missing rates were imputed inherently in the SKAT analyses by Hardy–Weinberg equilibrium. The Benjamini–Hochberg false discovery (FDR) adjusted P value was applied to correct for multiple testing. All statistical analyses were conducted using the statistical programing language R (version 3.3.1) [35] and the R packages SKAT (version 1.2.1) and mediation (version 4.4.6) [36, 37] was employed for the SKAT analysis and mediation analysis, respectively. All tests were 2-sided unless noted otherwise. A P-value less than 5% was considered statistically significant.

## Results

### Characteristics of study participants

Our study included a total of 364 women. Study participants were mostly non-Hispanic Whites (66.5%) and African Americans (28.3%) (Table 1). The median age was 48 (IQR: 44 ~ 51) years. The median body mass index (BMI) was 28.1 kg/m^2^ (IQR:24.1 ~ 34.9). The median age at first birth was 26 years (IQR: 21 ~ 30) years and 26.1% of the women had a family history of breast cancer. Medium serum progesterone level was 0.3 ng/mL (IQR 0.2 ~ 3.2). Median VPD was 7.3% (IQR:4.7 ~ 12.3) and BI-RADS® category b (scattered areas of fibroglandular density) was the most common (41.2%) mammographic breast density category.

**Table 1:** Characteristics of study participants (n = 364) recruited at the Joanne Knight Breast Health Center at Siteman Cancer Centre at Washington University School of Medicine, (St. Louis, MO) in 2016

Characteristic	Number	Median (IQR) or Count (%)*
**Age (years)**	364	48 (44 ~ 51)
**Progesterone (ng/mL)**	364	0.31 (0.2 ~ 3.2)
**Mammographic breast density**		7.3 (4.7 ~ 12.3)
Volumetric percent density (%)^a^	364	7.3 (4.7 ~ 12.3)
< 3.5%		34 (9.3)
3.5–7.5%		150 (41.2)
≥ 7.5–15.5%		116 (31.9)
≥ 15.5%		64 (17.6)
Non-dense volume (cm^3^)	364	865.65 (528.9 ~ 1411.3)
**Race**	364	
Non-Hispanic White		242 (66.5)
Black or African American		103 (28.3)
Others		14 (3.9)
Missing		5 (1.4)
**Age at first birth (years)**	295	26 (21 ~ 30)
**Age at second birth (years)**	228	29 (25 ~ 33)
**Parity and Age at first birth**	364	
Nulliparous		66 (18.1)
1–2 kids; < 25		63 (17.3)
1–2 kids; 25 ~ 29		46 (12.6)
1–2 kids; > = 30		78 (21.4)
> = 3 kids; < 25		60 (16.5)
> = 3 kids; > = 25		48 (13.2)
Missing		3 (0.9)
**BMI (kg/m**^**2**^**)**	364	28.13 (24.1 ~ 34.9)
Normal		113 (31.0)
Overweight		96 (26.4)
> = 30		148 (40.7)
Missing		7 (1.9)
**Body Shape at Age 10**	364	
1 ~ 2		136 (37.4)
3~4		108 (29.7)
5		45 (12.4)
> = 6		26 (7.1)
Missing		49 (13.5)
**Family history of breast cancer**	364	
Yes		95 (26.1)
No		257 (70.6)
Missing		12 (3.3)
**Phase of menstrual cycle**	364	
Follicular		78 (21.4)
Luteal		123 (33.8)
Oligomenorrhea		104 (28.6)
Missing		59 (16.2)

^a^Volumetric Percent Density. Volpara volumetric percent density ranges from 0.5 to 34.5%. Corresponding to the four categories (a) ~ (d) of the breast imaging reporting and data system (BI-RADS®) (5th edition), Volpara volumetric percent density measures translate to: < 3.5 (a—almost entirely fatty breasts); ≥ 3.5 and < 7.5 (b—scattered areas of fibroglandular density); ≥ 7.5 and < 15.5 (c—heterogeneously dense breasts); ≥ 15.5% (d—extremely dense breasts)

^*^Summary include median and interquartile range (IQR) for quantitative variables and count (%) for categorical variables

### Correlations between circulating progesterone, volumetric percent density, and non-dense volume

**Fig. 2:**
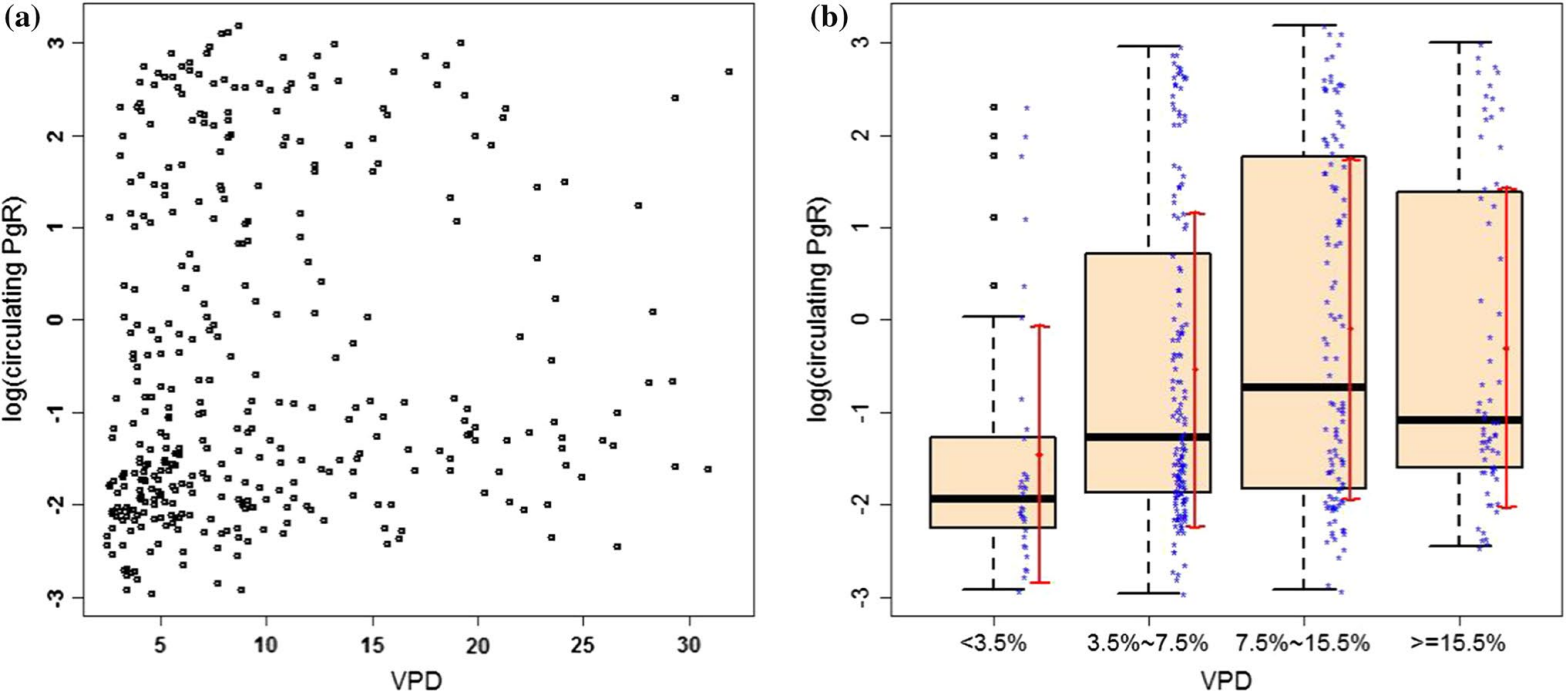
**a** Scatterplot of cPgR (in log scale) with VPD (Spearman cor = 0.21, p = 5.35E-05) **b** Boxplot of PgR (in log scale) by VPD category (blue points represent each data point, red dot and arrow indicate mean and SD, black horizontal line indicates median). The overall Kruskal–Wallis test comparing circulating PgR by the 4 VPD categories indicated significant difference among the 4 categories (P = 9.58E-05) and post-hoc pairwise Wilcoxon rank sum test with Benjamini–Hochberg adjustment on p values indicated significant difference between the lowest “ < 3.5%” category with each of the three higher categories in sequential order (adjusted P = 0.00074, 0.00011, and 0.00011, respectively) but not others

VPD and NDV were negatively correlated (Spearman correlation = -0.78, P < 2.2E-16). circulating progesterone was weakly positively correlated with VPD (Spearman correlation = 0.21, P = 5.34e-05, Fig. 2a).

and negatively correlated with NDV (Spearman correlation = − 0.19, P = 0.0003). When stratified by VPD categories, the lowest level of circulating progesterone (median, IQR = 0.14, 0.11 ~ 0.26) was observed in the lowest VPD category (VPD ≤ 3.5%) compared with each of the three higher categories (median, IQR = 0.28, 0.16 ~ 1.98, adjusted P = 0.00074 for 3.5% ≤ VPD < 7.5%; and = 0.49, 0.16 ~ 5.66, adjusted P = 0.00011 for 7.5% ≤ VPD < 15.5%; = 0.34, 0.20 ~ 3.89, adjusted P = 0.00011for VPD ≥ 15.5%, respectively, , Online Resource 1).

### Associations of SNP sets with circulating progesterone, volumetric percent density, and non-dense volume

After initial QC filtering, 162 SNPs were available for analyses; 140 for PGR and 22 for PGRMC1 (see Online Resource 2 for SNP information and genotype count and frequency). We used the SKAT approach to test the overall associations between circulating progesterone of the SNP set on both PGR and PGRMC1 and each gene alone. In univariate analysis, PGR pathway SNPs (from both PGR and PGRMC1) were collectively associated with VPD (SKAT P = 0.013) and NDV (SKAT P = 9.46E-05) but not circulating progesterone (SKAT P = 0.473) (Online Resource 1). The significant association with VPD was contributed by SNPs in the PGR gene (SKAT P = 0.017) but not SNPs in the PGRMC1 gene (SKAT P = 0.113). Both PGR SNPs and PGRMC1 SNPs were each associated with NDV (PGR: SKAT P = 0.00026; PGRMC1: SKAT P = 0.006) and neither with circulating progesterone (PGR: SKAT P = 0.390; PGRMC1: SKAT P = 0.846). After adjusting for confounders, the SKAT analyses were not statistically significant for both PGR and PGRMC1.

### Associations of SNPs with circulating progesterone, volumetric percent density, and non-dense volume

**Fig. 3:**
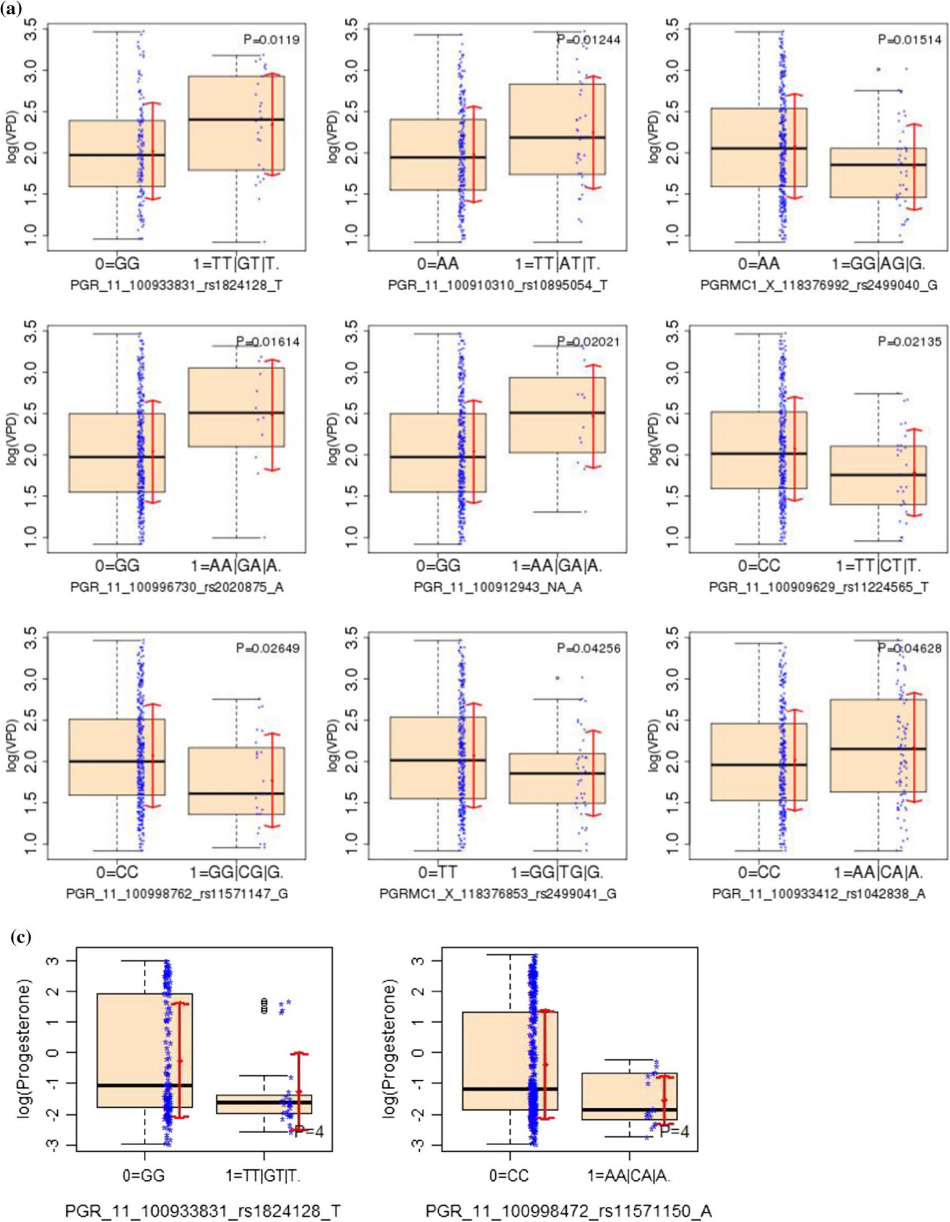

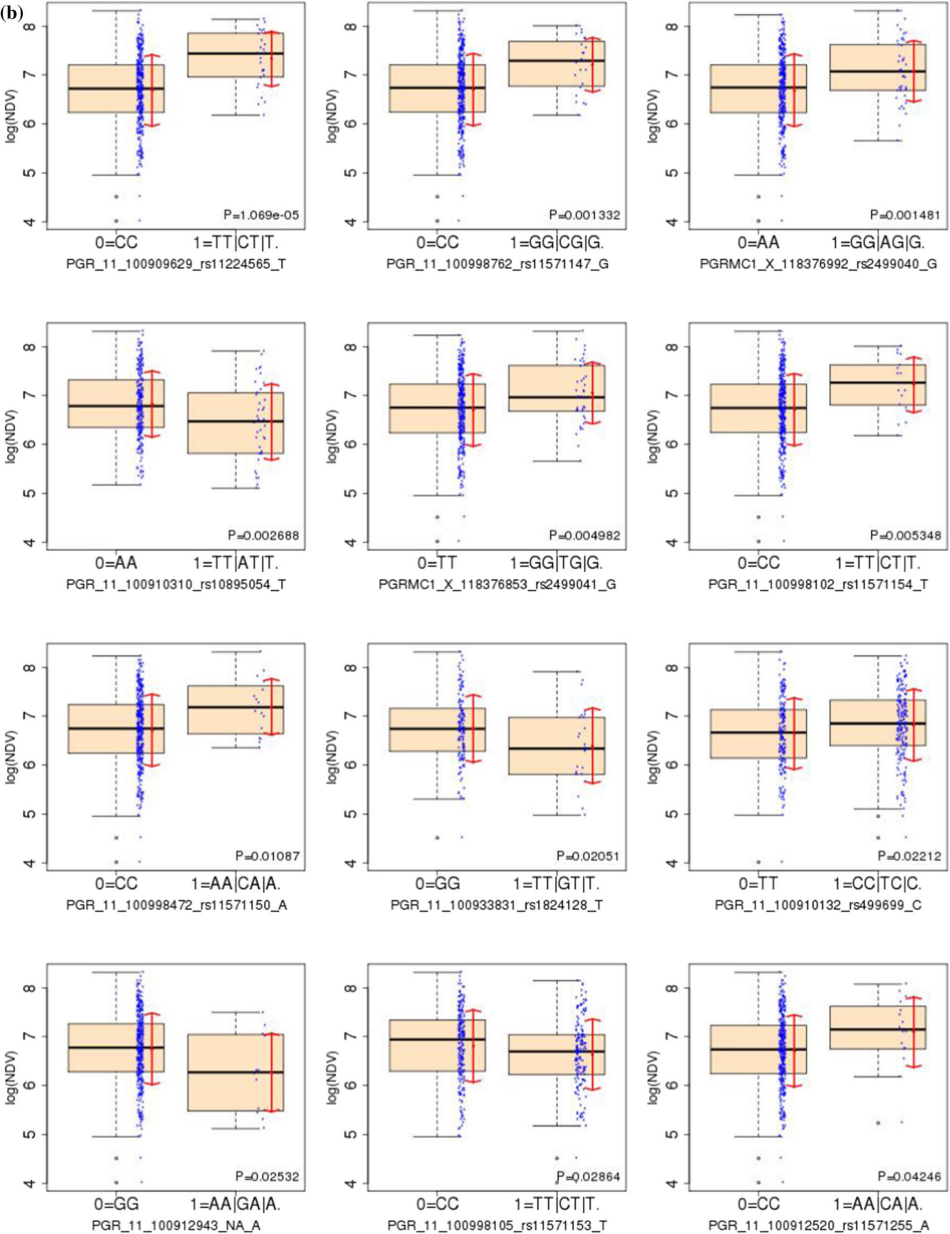
Single nucleotide polymorphisms (SNPs) associated with **A** volumetric percent density (VPD), B non-dense volume (NDV) and **C** circulating progesterone (cPgR) (all in log scale) from univariate linear regression

We investigated the associations of each SNP (with a genotype = 1 count >  = 10 and frequency ≥ 3%) with circulating progesterone, VPD, and NDV without (Table 2) and with (Table 3, Online Resource 3) adjustment for confounders. Fourteen SNPs, 12 on PGR and 2 on PGRMC1, were statistically significant with unadjusted p-values < 5% (Table 2; Fig. 3) in univariate analyses. Of these, PGR rs1824128 was significantly associated with VPD (estimate = 0.32, SE = 0.13, p-value = 0.012), NDV (estimate = -0.35, SE = 0.15, p-value = 0.021) and circulating progesterone (estimate = -1.02, SE = 0.38, p-value = 0.009). In multivariable analyses, one SNP (rs657516) for VPD, none for NDV and five SNPs for circulating progesterone had p-values < 0.05 but none was statistically significant after FDR correction with the FDR-corrected p-values of 0.08 for the 4 PGR SNPs (Table 3). Six SNPs: 4 on PGR (rs10895054, rs11224565, rs11571147, and rs11571154) and 2 on PGRMC1 (rs2499040, rs2499041) were significantly associated with NDV in univariate analyses after FDR correction (Table 2c), but not after adjusting for confounders.

**Table 2:** Single nucleotide polymorphisms (SNPs) with significant univariate associations (without adjustment for covariates) among 42 SNPs: with genotype 1 frequency >  = 3% and count >  = 10) with (a) circulating progesterone (cPgR), (b) volumetric percent density (VPD) and (c) non-dense volume (NDV) from linear regression

(a)						
SNP ID			cPgR			
Gene	SNP	Minor Allele	Estimate	SE	P value	FDR P-value
PGR11	rs1824128	T	− 1.02	0.38	0.009	0.209
PGR11	rs11571150	A	− 1.16	0.45	0.010	0.209

(b)						
SNP ID			VPD			
Gene	SNP	Minor Allele	Estimate	SE	P value	FDR P-value
PGR11	rs1824128	T	0.32	0.13	0.012	0.149
PGR11	rs10895054	T	0.26	0.10	0.012	0.149
PGRMC1	rs2499040	G	− 0.25	0.10	0.015	0.149
PGR11	rs2020875	A	0.44	0.18	0.016	0.149
PGR11	NA	A	0.43	0.18	0.020	0.149
PGR11	rs11224565	T	− 0.29	0.13	0.021	0.149
PGR11	rs11571147	G	− 0.30	0.13	0.027	0.159
PGRMC1	rs2499041	G	− 0.22	0.11	0.043	0.216
PGR11	rs1042838	A	0.15	0.08	0.046	0.216

(c)						
SNP ID			NDV			
Gene	SNP	Minor Allele	Estimate	SE	P value	FDR P-value
PGR11	rs1824128	T	− 0.35	0.15	0.021	0.103
PGR11	rs10895054	T	− 0.37	0.12	0.003	**0.028**
PGRMC1	rs2499040	G	0.39	0.12	0.002	**0.021**
PGR11	rs2020875	A	− 0.42	0.22	0.052	0.163
PGR11	NA	A	− 0.49	0.22	0.025	0.106
PGR11	rs11224565	T	0.64	0.14	0.000	**0.000**
PGR11	rs11571147	G	0.51	0.16	0.001	**0.021**
PGRMC1	rs2499041	G	0.35	0.12	0.005	**0.037**
PGR11	rs1042838	A	− 0.15	0.09	0.110	0.256
PGR11	rs11571255	A	0.38	0.19	0.043	0.149
PGR11	rs499699	C	0.18	0.08	0.022	0.103
PGR11	rs11571150	A	0.48	0.19	0.011	0.065
PGR11	rs11571154	T	0.51	0.18	0.005	**0.037**
PGR11	rs11571153	T	− 0.17	0.08	0.029	0.109

*Estimate: linear regression coefficient estimate; SE: standard error, P value: Wald test P; *FDR P* FDR adjusted P value across all SNPs.

**Table 3:** Single nucleotide polymorphisms (SNPs) with significant multivariate adjusted (for the six covariates) associations among 42 SNPs: with genotype 1 frequency >  = 3% and count >  = 10) with (A) circulating Progesterone (cPgR), (B) volumetric Percent Density (VPD)

(a)						
SNP ID			cPgR			
Gene	SNP	Minor Allele	Estimate	SE	P value	FDR P-value
PGR11	rs11571241	T	− 1.81	0.63	0.004	0.076
PGR11	rs11571239	T	− 1.81	0.63	0.004	0.076
PGR11	rs1824128	T	− 1.21	0.44	0.007	0.076
PGR11	rs11571150	A	− 1.55	0.57	0.007	0.076
PGRMC1	rs41294894	G	− 1.81	0.85	0.037	0.308

(b)						
SNP ID			VPD			
Gene	SNP	Minor allele	estimate	SE	P value	FDR P-value
PGR11	rs657516	G	− 0.1966	0.0952	0.0399	0.9565

^*^Estimate: linear regression coefficient estimate

*SE* standard error, *P value* Wald test P, *FDR P* FDR adjusted P value

### Mediation analyses of PGR SNPs, circulating progesterone with volumetric percent density, and non-dense volume

**Fig. 4:**
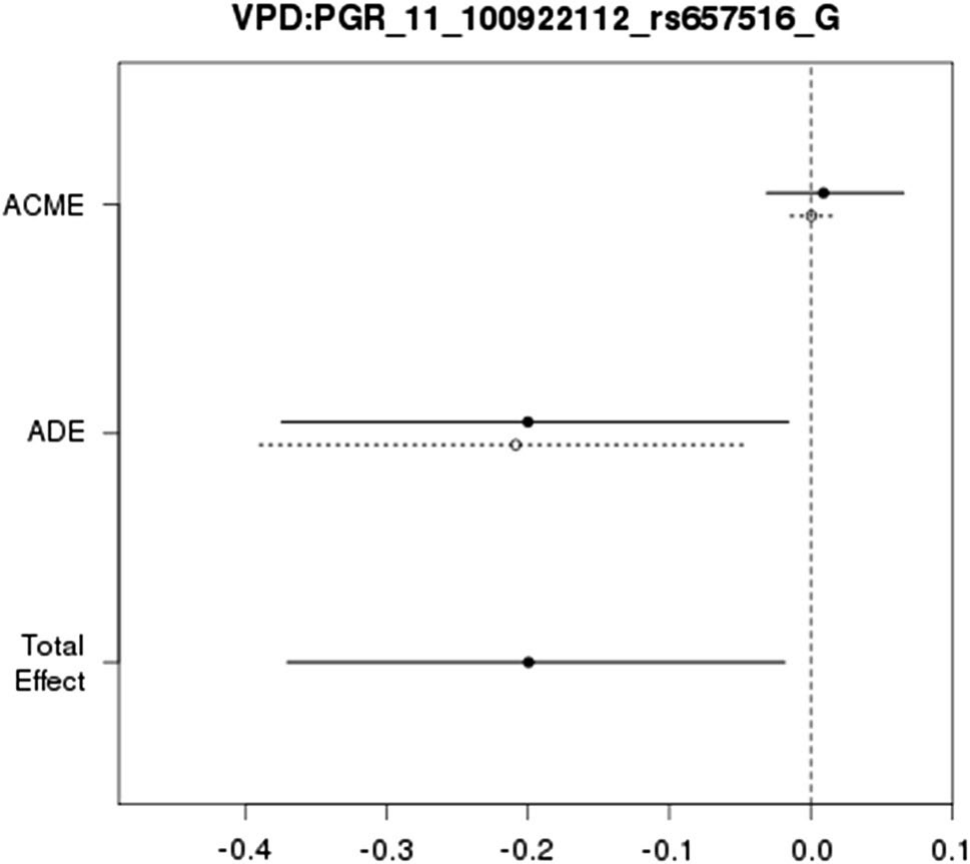
Direct effect (ADE), mediation effect (ACME) and total effect by genotype (solid line: genotype 1 of at least 1 minor allele “G”; dotted line: genotype 0 of two major alleles) of PGR_11_100922112_rs657516_G for VPD

To investigate whether circulating progesterone mediates the associations of PGR SNPs with VPD and NDV, we performed mediation analyses focusing on the 42 SNPs with genotype = 1 count >  = 10 and frequency > 3%. After adjustment for confounders, only PGR rs657516 had a direct effect on VPD (averaged direct effect estimate = − 0.20, 95% CI = − 0.38 ~ − 0.04, P = 0.02, Fig. 4) with additional adjustment for circulating progesterone and interaction between SNP and circulating progesterone besides the six covariates and the effect was homogeneous between the two genotypes, but there was no mediation effect (mediation effect averaged across the two genotypes = 0.01, 95% CI = − 0.02 ~ 0.03, P = 0.704).

### Associations of SNPs with determinants of mammographic breast density

Many SNPs were associated with the determinants of MD in multivariable analyses but most were not statistically significant after FDR correction (Online Resource 4, Online Resource 5). Four SNPs: 3 on PGR (rs11224565, rs10895054, and rs11571147) and 1 on PGRMC1 (rs2499040) were associated with BMI after FDR corrections (Online Resource 4). The rs11571153 SNP was positively associated with body shape at age 10 (30.6% of women with at least 1 copy of the effect allele (T) had a larger shape with a body shape = 5 or >  = 6 versus 16.6% in the women without the effect allele, raw P = 0.016) (AOnline Resource 5).

## Discussion

SNPs in the PGR and PGRMC1 genes were not associated with circulating progesterone, VPD and NDV after FDR correction. Circulating progesterone did not mediate the associations of SNPs in PGR-related genes with MD, suggesting that the effects, if any, of these SNPs are independent of circulating progesterone. Our findings should, however, be interpreted in the context of the small sample size.

To the best of our knowledge, this is one of the first studies to investigate the associations of SNPs in PGR and PGR-related genes with MD in premenopausal women and determine whether circulating progesterone levels mediate these associations. Although there were no statistically significant associations after correcting for multiple testing, some SNPs are still worth discussing because of the associations observed prior to correcting for multiple testing. PGR rs657516 SNP had an inverse average direct effect on VPD. This SNP is located within the enhancer region (GH11J100921) on chromosome 11[38]. PGR is one of the genes regulated by this enhancer region [38]. The location of this variant at an enhancer region suggests that this SNP may affect gene expression, activity, or function of PGR. A substitution in the enhancer region for PGR may cause downregulation of PGR expression, reduce progesterone’s proliferative action in the breasts and explain the observed inverse direct effect on volumetric percent density. PGR rs1824128 was the only SNP in our study which was significantly associated with circulating progesterone, VPD and NDV. This SNP was positively associated with VPD and negatively associated with NDV and circulating progesterone and had previously been shown to be associated with an increased risk of breast cancer [39].

PGR SNPs rs2499041 and rs11571154 were significantly associated with NDV prior to FDR correction. Rs2499041 is a rare intronic variant with no known clinical significance [40, 41]. PGR SNP rs11571154 is also in the non-coding region [40, 42]. PGR rs11571150, which was associated with circulating progesterone is a missense variant with high frequency (MAF 0.12) in African Americans [40, 43]. PGRMC1 rs41294894 is an intron variant with increased frequency in persons of African descent (0.13) [40, 44]. This SNP was inversely associated with circulating progesterone. The inverse association of rs41294894 with circulating progesterone suggests that it could be associated with a reduction in breast cancer risk since serum progesterone has been associated with increased breast cancer risk [45] and deserves further evaluation in larger studies (Online Resource 6).

PGR SNPs rs11224565, rs10895054, rs11571147 and PGRMC1 rs2499040 were associated with BMI after FDR correction. Notably, all four SNPs were also associated with VPD and/or NDV in univariate analyses. These findings suggest a possible interaction between BMI and MD mediated through PGR-related gene variants. Our sample is, however, not large enough to evaluate these hypotheses, hence, larger studies are needed to characterize these. Rs11224565 is a non-coding variant located in the 3’UTR and could potentially influence BMI via alterations in gene expression [40, 46]. Prior work has shown that rs10895054 is associated with increased risk of breast cancer in African American women [47]. BMI at age 10 is inversely associated with breast cancer risk in premenopausal women [48]. Thus, the rs10895054 SNP may provide an insight into the associations of BMI with breast cancer risk in premenopausal women. The rs11571153 SNP [49] was positively associated with body shape at age 10, but inversely associated with NDV. This variant could play a role in early life body shape and explain in part the associations of adiposity at age 10 with MD in premenopausal women that we have previously reported [21].

Both salivary and circulating luteal phase progesterone levels are positively associated with MD in premenopausal women [50, 51]. Other studies have shown positive associations between endogenous progesterone levels and MD [52–56]. Prior work has also shown that women with PGR + 331 GG genotype were more susceptible to the effects of hormone therapy use on MD [19], however this study included both pre-, peri- and post-menopausal women. Our study focused on the associations between SNPs in PGR-related genes and MD in premenopausal women as well as the possible role that circulating progesterone plays in this association.

Our study has the following limitations which should be considered when interpreting the results: (i) the cross-sectional study design, (ii) our sample size was small and could explain why some results were null after FDR correction. The small sample size also precluded us from performing analyses stratified by race. In spite of these limitations, our study has the following strengths: (i) study participants were recruited among women attending annual routine screening mammogram, which enhances generalizability of our findings, (ii) we used Volpara to measure MD. Volpara provides automated, highly reproducible and robust volumetric measures of MD, (iii) we adjusted for the phase of the menstrual cycle in our analyses to account for the cyclical variation in circulating progesterone levels as circulating progesterone varies greatly by phase of menstrual cycles in premenopausal women.

In conclusion, SNPs in PGR-related genes (PGR and PGRMC1) were not associated with MD in this study population of 364 premenopausal women. Larger studies are needed to further investigate the intricate associations between progesterone signaling and MD in premenopausal women, and to confirm the associations of PGR-related SNPs with circulating progesterone and BMI.

## Supplementary Information

Supplementary file 1 (XLSX 10 KB).

Supplementary file 2 (XLSX 20 KB).

Supplementary file 3 (XLSX 19 KB).

Supplementary file 4 (XLSX 11 KB).

Supplementary file 5 (XLSX 15 KB).

Supplementary file 6 (XLSX 370 KB).

## Data Availability

The raw datasets generated during the current study are available from the corresponding author on reasonable request. All data analysed during this study are included in this published article [and its supplementary information files].
